# Stressed out: Bacterial response to high salinity using compatible solute biosynthesis and uptake systems, lessons from *Vibrionaceae*

**DOI:** 10.1016/j.csbj.2021.01.030

**Published:** 2021-02-01

**Authors:** Gwendolyn J. Gregory, E. Fidelma Boyd

**Affiliations:** Department of Biological Sciences, University of Delaware, Newark, DE 19716, United States

**Keywords:** Compatible solutes, Metabolism, Transporters, Ectoine, Glycine betaine, DMSP

## Abstract

Bacteria have evolved mechanisms that allow them to adapt to changes in osmolarity and some species have adapted to live optimally in high salinity environments such as in the marine ecosystem. Most bacteria that live in high salinity do so by the biosynthesis and/or uptake of compatible solutes, small organic molecules that maintain the turgor pressure of the cell. Osmotic stress response mechanisms and their regulation among marine heterotrophic bacteria are poorly understood. In this review, we discuss what is known about compatible solute metabolism and transport and new insights gained from studying marine bacteria belonging to the family *Vibrionaceae*.

## 1 Introduction

**Fig. 1:**
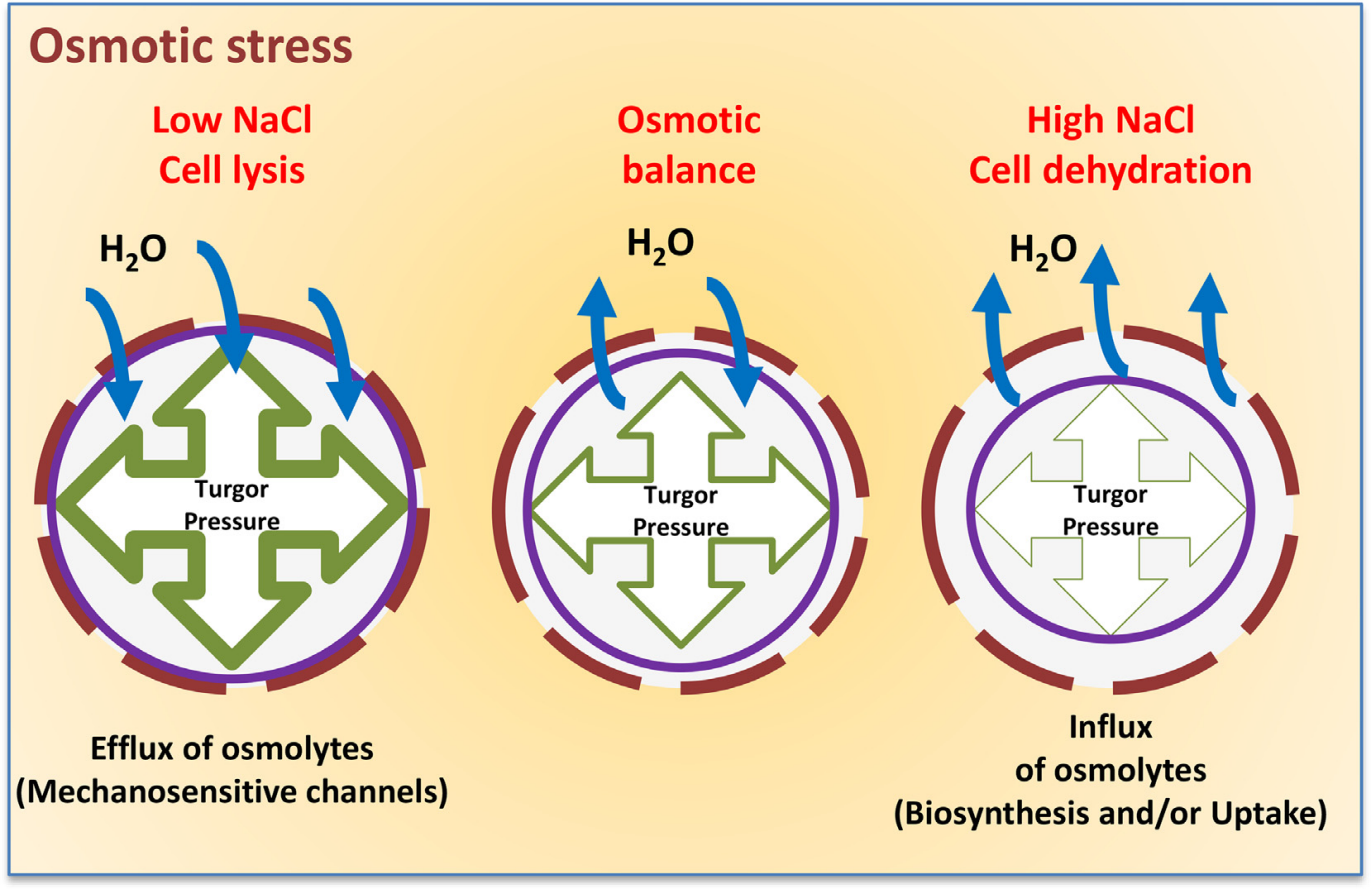
Osmotic stress and water flux into and out of cell. In low NaCl conditions water flows into cell causing increased turgor pressure, which is counteracted by removing osmolytes. In high NaCl conditions water flows out of cell and this is counteracted by accumulating osmolytes.

Halophilic and halotolerant bacteria in marine environments encounter a range of NaCl concentrations. Bacteria that live in these environments have adapted to grow optimally in high salinity and to cope with fluctuations in salinity to maintain cellular homeostasis (Fig. 1). Growth of bacteria in high salinity lowers the turgor pressure of the cell due to efflux of water across the osmotic gradient (Fig. 1) [1], [2], [3], [4], [5]. To combat this loss of turgor pressure in hyper-osmotic conditions, bacteria have developed strategies for osmotic stress adaptation [1], [2], [3], [4], [5], [6]. Extreme halophiles from the domain Archaea use the salt-in cytoplasm response that results in the accumulation of inorganic ions in the cytoplasm in high molar concentrations. In the domain Bacteria, osmoadaptation occurs in two stages exemplified in the Gram-negative and Gram-positive bacteria *Escherichia coli* and *Bacillus subtilis,* respectively [7], [8], [9], [10], [11], [12]. The initial and short-term response is characterized by the uptake of potassium (K^+^) ions in response to increased external osmolarity. The strong positive charge of the K^+^ ions must be balanced to prevent damage to biological molecules and processes. Gram-negative bacteria biosynthesize or uptake from the environment organic anions, such as glutamate, to counterbalance the charge of the accumulating K^+^ [1], [2], [3], [4], [5], [6], [13]. In contrast, the counter ion that balances K^+^ accumulation in Gram-positive bacteria is unknown, as intracellular glutamate only minimally increases or decreases. High concentrations of K^+^ have deleterious effects on cellular processes and accumulation is a short-term strategy. The secondary long-term strategy involves the uptake and/or biosynthesis of compatible solutes, which are also referred to as osmolytes. It was proposed that accumulated K^+^ and glutamate act as a trigger of this secondary long-term response [1], [2], [3], [4], [5], [6], [13]. This was demonstrated in the halophilic bacterium *Halobacillus halophilus*, where K^+^ and glutamate accumulation induces biosynthesis of the compatible solute proline [14].

### 1.1 Range of compatible solutes

**Fig. 2:**
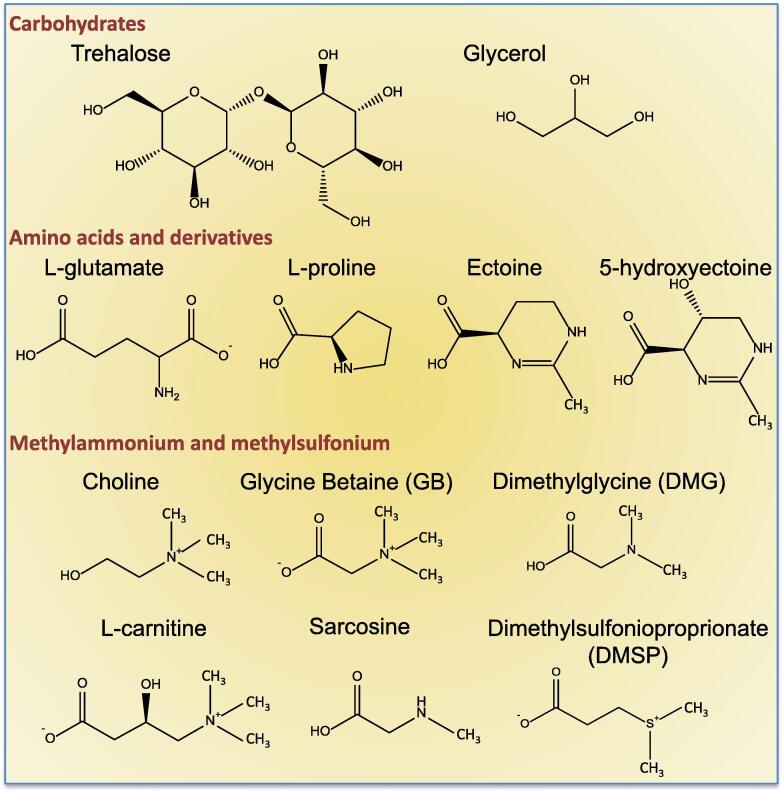
Structures of common compatible solutes. Examples of structural categories of compatible solutes include: trehalose (sugar), glycerol (polyol), glutamate and proline (free amino acids), ectoine and hydroxyectoine (amino acid derivatives), glycine betaine (GB), and carnitine (quarternary amines), dimethylglycine (DMG, tertiary amine), and dimethylsulfoniopropionate (DMSP, tertiary sulfonium).

Compatible solutes are a restricted group of low molecular weight compounds that can be classified into structural categories examples of which include trehalose (a sugar), glycerol and mannitol (polyols), L-proline, L-glutamate and L-glutamine (free amino acids), ectoine and 5-hydroxyectoine (amino acid derivative), glycine betaine, L-carnitine (quaternary amines), dimethylglycine (tertiary amine), choline-*O*-sulfate (sulfate ester), dimethylsulfoniopropionate (tertiary sulfonium), and N-acetylated diamino acids among others (Fig. 2) [1], [2], [3], [4], [5], [6], [13]. Bacteria can accumulate compatible solutes in molar concentrations without affecting the molecular processes of the cell. This accumulation also causes an increase in free water in the cell, which is a major determining factor in growth and division in hyper-osmotic conditions [4], [5]. Compatible solutes thus allow cells to continue to grow and divide in unfavorable environments. It has been shown that compatible solutes also protect proteins, nucleic acids and other vital molecular machinery by increasing the hydration shell around these molecules [1], [2], [3], [4], [5], [6], [13]. Accumulation of compatible solutes can be accomplished either by transport into the cell or biosynthesis. There are only a few examples of *de novo* compatible solute biosynthesis pathways with most pathways requiring an exogenous precursor. Uptake of osmolytes can be easily accomplished using a variety of transporters, which include primary transporters from the ATP-binding cassette (ABC) family that require ATP, and secondary transporters of the Betaine Carnitine Choline Transporter (BCCT) family, the Major facilitator superfamily (MFS) and the tripartite ATP-independent periplasmic (TRAP) family of transporters [15], [16], [17], [18], [19], [20], [21], [22], [23], [24], [25]. Bacterial species can biosynthesize L-proline, L-glutamate, L-glutamine and trehalose in response to osmolarity changes. However, these compounds are also utilized in different metabolic pathways in the cell and as carbon sources, therefore their use as osmolytes in some species can be an energy sink [4], [23], [26].

### 1.2 Vibrionaceae, marine species with diverse lifestyles

The family *Vibrionaceae*, members of the sub-phylum *Gamma-Proteobacteria*, are cosmopolitan in their distribution in marine environments, present in salt marshes, deep-sea sediments, throughout the water column and associated with marine flora and fauna. *Vibrionaceae* are the most significant consumers of chitin (β1,4-linked N-acetylglucosamine), the second most abundant polymer in the ocean [27], [28], [29]. Estimates of chitin production range from ~10^10^ to 10^11^ tons globally, which is rapidly recycled by *Vibrionaceae*, highlighting the importance of this group to ocean health and their capacity to flourish in marine environments [30]. Members of the *Vibrionaceae* exemplify the range of osmo-adaptation mechanisms marine bacteria have evolved to counter fluctuations in osmolarity to prevent either dehydration or rupture of the cell under hyper-osmotic and hypo-osmotic conditions, respectively. There are several described genera within the *Vibrionaceae* including *Aliivibrio* (formerly *Vibrio*), *Enterovibrio, Grimontia, Photobacterium, Salinivibrio,* and *Vibrio* that have significant genome data available [31], [32], [33], [34]*.* The genus *Vibrio* is probably the most well studied as the causative agents of pandemic infectious diseases in humans specifically, *V. cholerae* and *V. parahaemolyticus*. In addition, many species are pathogens of corals, fish, and shellfish and cause huge economic loses to the aquaculture industry. *Vibrio* species are also important model organisms for the study of biofilm formation, natural competence, symbiosis, and quorum sensing pathways [31], [32], [33], [34]. Members of the *Vibrionaceae*, unlike most *Proteobacteria*, contain a divided genome with two circular chromosomes of unequal size. The genome size among *Vibrionaceae* ranges from approximately 4.0 Megabases (Mb) to just under 6.0 Mb, with the differences in size usually present in chromosome II. A majority of the genes required for core cell maintenance and survival (replication, transcription, and translation) are present on chromosome I and many genes encoding phenotypes that differentiate strains and species are present on chromosome II. Since the first whole genome sequence of *Vibrio cholerae* was reported in 2000 followed by *V. parahaemolyticus* in 2003, the advent of next generation sequencing (NGS) has resulted in an exponential growth of available genome sequences. As of November 2020, there were 9,544 genome assemblies of *Vibrionaceae* deposited in the NCBI genome database, 8,712 of which were *Vibrio* species. Many species within the family *Vibrionaceae* are halophiles and have adapted to grow optimally in high salinity conditions. *Vibrio* species have been shown to biosynthesize and rapidly import a diverse range of compatible solutes making them an ideal model to study osmotic stress systems [20], [31], [32], [35], [36], [37].

In this review, we will give an overview of known compatible solutes utilized by bacteria, and their metabolism and transport pathways*.* We will discuss what can be gleaned from genome mining regarding the identification of genes required for ectoine, 5-hydroxyectoine, glycine betaine (GB), dimethyl glycine (DMG), sarcosine and dimethylsulfoniopropionate (DMSP) metabolism in members of the *Vibrionaceae*. The type and diversity of osmolyte transporters and the variety of substrates these carriers can accommodate in *Vibrio* will also be discussed. Finally, we will discuss the mechanisms of regulation of compatible solute systems in *Vibrio*, specifically the more recent discoveries regarding the role of quorum sensing regulators (AphA and OpaR) and global regulator CosR.

## 2 Compatible solutes metabolism and transport in bacteria

In this section, an overview will be given of the *de novo* biosynthesis pathways of compatible solutes such as ectoine and 5-hydroxyectoine as well as the biosynthesis of methylammonium and methylsulfonium compounds GB, DMG, and DMSP in bacteria (Fig. 2). Additionally, we will discuss the use of compatible solutes as rich nutrient sources and the catabolic pathways present among bacteria. An outline of the major classes of compatible solute transporters in bacteria will be provided.

### 2.1 Ectoine and 5-hydroxyectoine metabolism

**Fig. 3:**
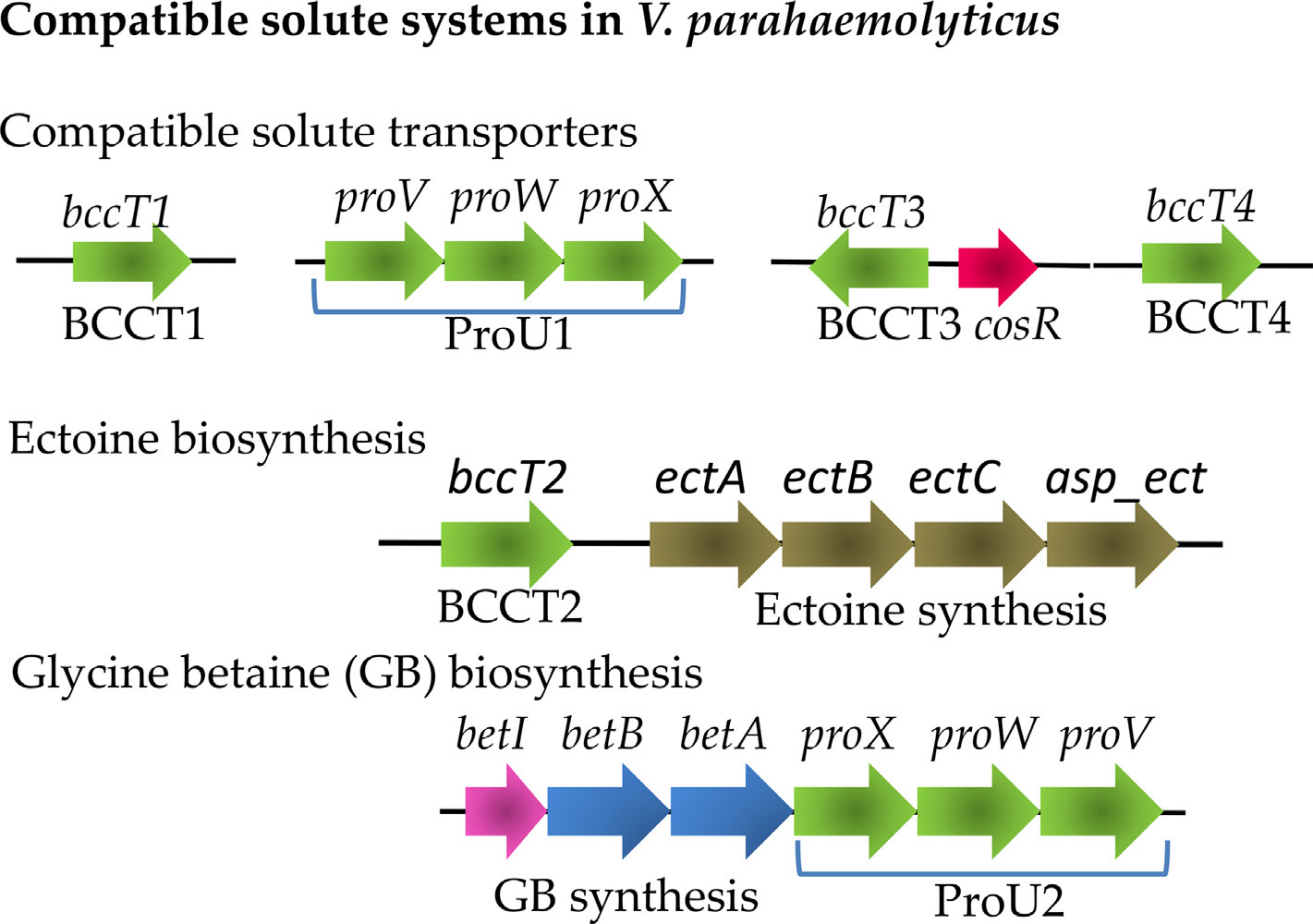
Compatible solute systems in *V. parahaemolyticus*. To date two compatible solute biosynthesis systems have been described in this group and at least six dedicated compatible solute transporters are known. Arrows indicate ORFs and direction of transcription, green arrows indicate transporters. (For interpretation of the references to colour in this figure legend, the reader is referred to the web version of this article.)

Ectoine (1,4,5,6-tetrahydro-2-methyl-4-pyrimidinecarboxylic acid) is biosynthesized *de novo* from endogenous cellular L-aspartic acid. EctA, EctB, and EctC, encoded by the operon *ectABC*, convert L-aspartic acid to ectoine and this operon is evolutionarily conserved in Gram- negative and Gram-positive bacteria [36], [38], [39], [40], [41], [42]*.* Several species that produce ectoine also encode a specialized aspartokinase (Ask) specific to the ectoine biosynthesis pathway and the gene involved clusters with the *ectABC* genes (Fig. 3) [36], [38], [39], [40], [41], [42]. For example, the halophile *V. parahaemolyticus* and other *Vibrio* species encode a specific aspartokinase (Asp_ect) in the same operon as the *ectABC* genes [36], [39], [43]. Ask/Asp_ect converts L-aspartic acid to β-aspartyl phosphate, which is then converted to L-aspartate-β-semialdehyde by aspartate semialdehyde dehydrogenase (Asd). This intermediate product in the L-aspartic acid pathway is then funneled into the ectoine biosynthesis pathway and converted to L-2,4-diaminobutyrate by the transaminase EctB. This product is then acetylated by EctA, and L-ectoine synthase (EctC) performs the cyclic condensation reaction to produce tetrahydropyrimidine ectoine, or ectoine [44]. The ectoine derivative 5-hydroxyectoine is also an osmolyte, and its biosynthesis requires an additional enzyme, ectoine dioxygenase (EctD), which converts ectoine to 5-hydroxyectoine [44], [45], [46]. EctD is present in some members of *Proteobacteria* and in Terrabacteria such as *Bacillus* and *Actinobacteria* species. In most genera, the *ectD* gene, in general, is clustered with *ectABC* [42], [44]. Ectoine was suggested as the main osmolyte produced by aerobic heterotrophic bacteria [36], [38], [39], [41], [47], [48], [49]. A bioinformatics study showed that ectoine biosynthesis is present predominantly in *Bacteria* and only a few *Archaea*. Among 6,428 microbial genomes examined, 440 species (7%) had ectoine biosynthesis genes and of these, 272 were predicted to synthesize 5-hydroxyectoine as well [42], [44].

Some bacteria can use ectoine and 5-hydroxyectoine as carbon and energy sources and also as a nitrogen source. Surprisingly, the genes required and the pathways used to catabolize ectoine and 5-hydroxectoine are still poorly understood [42]. The minimal requirement for ectoine degradation has been proposed to consist of an ectoine hydrolase (EutD/DoeA) and deacetylase (EutE/DoeB) [50]. The genes encoding these enzymes were first identified in *Sinorhizobium meliloti* and were present in the operon *eutABCDE* that also include *eutABC* required for 5-hydroxyectoine degradation to ectoine. In addition, an ABC transporter encoded by *ehuABCD* and a regulator in the GntR superfamily encoded by *enuR* are also part of the same gene cluster [42], [50], [51], [52]. Divergently transcribed from these genes are *asnR-ssd-atf-nqr* that encode an AsnC/Lrp family regulator, a succinate semialdehyde dehydrogenase, an aspartate aminotransferase, and an oxidoreductase, respectively. It was shown that these genes were induced by ectoine and 5-hydroxyectoine. A study in *Halomonas elongata* DSM 2581 T demonstrated that ectoine degradation began with the hydrolysis of the ectoine ring that ultimately resulted in the formation of L‐aspartic acid [53]. In the marine bacterium *Ruegeria pomeroyi*, which can only consume ectoine, it was proposed that the EutD hydrolase homodimer generates N-α-2 acetyl-L-2,4-diaminobutyrate (α -ADABA) from ectoine that is then deacetylated by the zinc-dependent EutE monomer into DABA, which is further catabolized to L-aspartate [54]. Of note is the finding that EutD can also hydrolyze 5-hydroxyectoine to form hydroxy– α -ADABA [54]. Using EutD as a query sequence in BLAST analysis, it was shown that this protein was confined to Proteobacteria, mainly *Alpha-, Beta-* and *Gamma-Proteobacteria* species [42], [54]. Some of the species that can catabolize ectoine and 5-hydroxyectoine can also biosynthesize them suggesting complex regulatory mechanisms controlling the two processes [54]. For a comprehensive review of the biochemistry, phylogenomics, and regulation of ectoine and 5-hydroxyectoine metabolism see Bremer and colleagues reviews [42], [44].

### 2.2 Glycine betaine (GB) and dimethylglycine (DMG) metabolism

The main function of choline (trimethyl-β-hydroxyethylammonium) in most species is as a metabolic precursor for glycine betaine (*N*,*N*,*N*-trimethylglycine) biosynthesis. Although, in *Pseudomonas syringae*, a plant pathogen, choline has been demonstrated to be a more potent osmoprotectant than GB [55]. Choline is imported into the bacterial cells by many different transporters, depending on the species [7], [15], [18], [19], [20], [55], [56], [57], [58], [59]. The biosynthesis of GB from choline is widespread among prokaryotes, halophilic phototrophic bacteria and archaeal methanogens [1], [13], [20], [32], [60], [61], [62]. Production of GB takes place in a two-step oxidation from the precursor choline and is not a *de novo* biosynthetic reaction. True *de novo* biosynthesis of GB is rare and proceeds through the methylation of glycine, a pathway identified in a limited number of bacteria [1], [61], [63], [64], [65], [66], [67]. Most bacteria generate GB via the oxidation of choline by the products of two genes *betB* and *betA*, which encode betaine-aldehyde dehydrogenase and choline dehydrogenase, respectively (Fig. 3). In *E. coli*, these genes are encoded by the operon *betIBA; betI*, is a repressor of its own operon and this repression is relieved in the presence of choline [7], [56], [57], [68], [69]. In *E. coli*, adjacent to the *betIBA* operon is *betT,* which encodes a BCCT transporter that uptakes choline. Choline is oxidized by the choline dehydrogenase BetA to produce betaine aldehyde, which is then oxidized to GB by BetB [8], [9], [57], [58]. In Gram-positive bacteria such as *Bacillus*, the choline-to-betaine aldehyde reaction uses an alcohol dehydrogenase. A second operon involved in GB biosynthesis from the precursor choline-O-sulfate, the *betICBA* operon, is restricted to *Alpha-Proteobacteria* within the family *Rhizobiaceae*, with *betC* encoding a choline sulfatase [70].

Several species have been shown to catabolize GB as a nutrient source in the genera Corynebacterium, Pseudomonas and Sinorhizobium [71], [72], [73]*.* However, only in *Pseudomonas* species has the complete pathway been described [71], [72], [73]. In this genus, serial demethylation of GB occurs with GbcA and GbcB, a dioxygenase and ferrodoxin reductase respectively converting GB to DMG. DgcAB demethylases then convert DMG to sarcosine (monomethylglycine), and finally SoxBDAG, a heterotetrameric sarcosine oxidase, converts sarcosine to glycine [71], [72], [73], [74], [75]. This pathway is also present in *Burkholderia thailandensis*, but the genes involved have a very different genome arrangement [76].

*N*,*N*-dimethylglycine (DMG) is an osmolyte that can be transported into cells and is utilized by several bacterial species as an osmo- and thermo-protectant [77], [78]. DMG is produced as an intermediate compound during *de novo* GB biosynthesis from glycine that also produces the monomethylglycine sarcosine (*N*-methylglycine) [79]. As stated above, *de novo* GB biosynthesis is rare among aerobic heterotrophic eubacteria [65], [66]. However, in halophilic phototrophic eubacteria and methanogens, such as *Methanohalophilus portucalensis*, betaine is *de novo* biosynthesized from glycine by methylation reactions with S-adenosylmethionine as methyl donor, and sarcosine and DMG serve as the intermediates [65], [66]. DMG is also an intermediate compound produced in the catabolism of GB, as described above [71], [72], [73]. In *Chromohalobacter salexigens*, DMG degradation to sarcosine was demonstrated, however, DMG was shown to inhibit growth rather than elicit osmo-protective affects [80].

### 2.3 Dimethylsulfoniopropionate (DMSP) metabolism

DMSP is an organosulfur compound abundant in marine surface waters that until recently was believed to be produced predominantly by phytoplankton, algae, and some halophytic vascular plants [81], [82], [83], [84], [85], [86]. DMSP is produced in vast quantities in the marine environment and used by phytoplankton, corals, and algae as an osmoprotectant, antioxidant, cryoprotectant, and a signaling molecule [87], [88], [89]. DMSP and its degradation product dimethyl sulfide (DMS) play important roles in global sulfur cycling and are also significant marine nutrients [89]. DMS is a climate active gas that is produced from the degradation of oceanic DMSP, releasing sulfur-containing aerosols into the atmosphere that aid the formation of cloud nuclei. The biosynthesis pathways of DMSP are poorly understood, however, recent studies have shown that marine heterotrophic bacteria can biosynthesize DMSP. These bacteria generate DMSP from the precursor methionine (Met) using a Met transamination pathway similar to that present in phytoplankton and algae [88], [89], [90], [91], [92], [93], [94], [95], [96], [97]. A key enzyme in this pathway, DsyB, a methylthiohydroxybutyrate methyltransferase, is present mainly among *Alpha-Proteobacteria* (Pseudooceanicola and Roseovarius). A methylation pathway for DMSP biosynthesis in Bacteria has also been identified. In this pathway, the key enzyme MmtN, a Met methyltransferase, is less prevalent than DsyB among bacteria. It is noteworthy that recent studies suggest DMSP-producing bacteria in coastal sediments may generate more DMSP than that produced in surface seawater by phytoplankton [92], [93], [95], [96].

The use of DMSP as a compatible solute for bacteria has been suggested but no comprehensive study has determined whether this is a prevalent phenotype among bacteria [98], [99], [100], [101], [102]. Although studies have shown that DMSP is assimilated by cyanobacteria *Prochlorococcus* and *Synechococcus* [103], [104]. In *S. meliloti*, it was shown that DMSP accumulated as a compatible solute and repressed the build-up of endogenous osmolytes in stressed cells [105]. Additional evidence that DMSP can be used as an osmoprotectant came from studies in *E. coli*, where it was shown that osmotically stressed cells responded to DMSP [102], [106]. The ABC-family ProU transporter was required for DMSP uptake in this species [102]. *Bacillus subtilis* was also shown to utilize DMSP to relieve osmotic stress and in *Bacillus* species, DMSP uptake was via the ABC-family transporters OpuC and OpuF [101], [107].

A restricted group of bacteria are able to catabolize DMSP using two pathways; a demethylation pathway and a cleavage pathway [98], [108], [109], [110], [111], [112], [113], [114], [115], [116]. The demethylation pathway results in the production of the volatile sulfur gas methanethiol (MeSH) and the cleavage pathway produces DMS and either 3-hydroxypropionate or acrylate [98], [108], [109], [110], [111], [112], [113], [114], [115], [116]. The DMSP lyases involved in these pathways are proteins that contain diverse domains and sequences. It is suggested that DMSP lyases have evolved by functional adaptation of existing proteins to exploit DMSP abundance and are a recent bacterial innovation. DMSP-catabolizing bacteria are present mainly in the sub-phylum *Alpha-Proteobacteria*; SAR11, SAR116 and *Roseobacter* [114], [116], [117], [118], [119], [120], [121]. However, recent data from *in situ* studies suggest that members of the sub-phylum *Gamma-Proteobacteria* also play a role in DMSP degradation [86], [121], [122], [123].

### 2.4 L-carnitine metabolism

L-carnitine (γ-trimethylamino-β-hydroxybutyric acid) is a quaternary amine compound present in muscle tissue. It is utilized by bacteria as an osmoprotectant as well as a thermoprotectant, however, there is no example of *de novo* synthesis of this compound by bacteria [124]. Depending on the species, L-carnitine is imported into the cells by an ABC transporter, BCCT or MFS carriers [15], [124], [125], [126], [127]. Like choline, in many species L-carnitine is converted to GB in a multistep process by CdhC, CdhAB, and DhcAB [15], [124], [125], [126], [127]. The catabolism of L-carnitine and choline has been reported and results in the production of trimethylamine (TMA) [124], [128]. TMA plays an important role in human health as it is associated with cardiovascular disease. Human gut microbiota can generate TMA from choline under anaerobic conditions [128]. The choline degradation pathway and gene cluster (*cut*) involved was recently described in *Desulfovibrio alaskensis* G20, a sulfate-reducing bacterium [128]. L-carnitine can also be metabolized to TMA) and malic semialdehyde by the oxidoreductase CntAB. TMA can be converted by flavin monooxygenase (FMO) to TMAO, which is also associated with heart disease. In bacteria, TMAO is an effective compatible solute for many species [124].

### 2.5 Trehalose metabolism

Trehalose is a compound used by a range of bacteria as an osmo- and thermo-protectant and bacteria can biosynthesize trehalose using at least three pathways; OtsAB, TreYZ and TreS [129], [130], [131]. The OtsAB pathway requires the precursors UDP-glucose and glucose-6-P, TreYZ uses maltodextrins as precursors and TreS uses the precursor maltose. Trehalose biosynthesis has not been described in *Vibrionaceae*, and homolog searches in the NCBI genome database did not identify these pathways in *Vibrionaceae*. However, *Vibrio* can uptake and use trehalose as a compatible solute and as a carbon source [77], [132]*.* Mannitol can also be biosynthesized for use as a compatible solute, but it biosynthesis is rare among bacteria [133], [134], [135]. Mannitol is produced from the precursor fructose‐6‐phosphate in a one-step reaction by mannitol‐1‐phosphate dehydrogenase or via a two-step pathway by mannitol‐1‐phosphatase [134], [135]. Bioinformatics analysis using these proteins as query sequences did not identify any homologs in *Vibrionaceae* genomes presence in the NCBI database.

### 2.6 Compatible solutes transporters

It is more energetically favorable to the cell to uptake compatible solutes from the environment rather than to biosynthesize them [4], [23], [26]. Therefore, Bacteria and Archaea encode numerous osmoregulated transporters to uptake compatible solutes with high affinity [15], [16], [18], [19], [20], [23]. One family of compatible solute transporters is the betaine-carnitine-choline transporter (BCCT) family; the founding member, BetT, was first discovered in *E. coli* and transports choline with high-affinity [57]. Other members of the BCCT family include an L-carnitine transporter, CaiT, present in *E. coli*, and GB transporters in *B. subtilis* (OpuD) and *Corynebacterium glutamicum* (BetP), among many others [24], [136], [137], [138], [139], [140]. The BCCT family uses a proton- or sodium-motive force to transport substrates into the cell [25]. The BCCT protein is organized into 12 transmembrane domains (TM to TM12), with TM8 comprised of a stretch of tryptophan residues, thought to be involved in subtrate binding [21], [141]. BCCTs have been shown to be induced in high osmolarity conditions [142]. For a comprehesive treatise on BCCTs structure and function in bacteria see Zeigler, Bremer and Kramer [25].

Bacteria and Archaea also utilize osmoregulated ABC transporters to import exogenous compatible solutes into the cell [18], [22], [51], [107], [143]. Examples of ABC-type transporters include ProU in *E. coli*, OpuA in *Lactococcus lactis*, OpuA, OpuB and OpuC in *B. subtilis*, and OpuC in *Pseudomonas syringae* [18], [22], [51], [107], [125], [143]. The ABC transporters are made up of a transmembrane domain (TMD), a nucleotide-binding domain (NBD) and a substrate-binding protein (SBP). Several ABC transporters contain a cystathionine-β-synthase (CBS) on the C-terminus of the NBD. The CBS domain was shown to be required for osmoregulatory function in *P. syringae* [144]. The NBD is encoded by the *proV* gene, while *proW* encodes the TMD and *proX* encodes the SBP [125], [144]. The *E. coli* ProU was shown to bind GB with high affinity and to be osmoregulated [145], [146], [147].

The third class of transporters known to uptake compatible solutes is the major facilitator superfamily (MFS) family, which are a large, diverse group of secondary transporters that includes uniporters, symporters, and antiporters. The MFS transporters possess either 12 or 14 transmembrane α-helical spanners, 6–7 in the N-terminus and 6–7 in the C-terminus linked by a center loop. These transporters are present in all domains of life and can transport a range of substrates into and out of the cell [148]. The classical example of an MSF osmolyte transporter is ProP from *E. coli*, an L-proline and GB transporter [148].

Lastly, tripartite ATP-independent periplasmic (TRAP) family of transporters have also been shown to uptake compatible solutes into the bacterial cell. A TRAP transporter TeaABC is present in *Halomonas elongata*, and is an osmotically inducible transporter for ectoine and 5-hydroxyectoine [149]. A TRAP transporter encoded by the *uehABC* genes has also been associated with ectoine and 5-hydroxyectoine uptake for use as a nutrient source and clusters with the ectoine and 5-hydroxyectoine catabolism genes in the marine bacterium *Ruegeria pomeroyi* DSS-3 [50].

## 3 Compatible solute systems in *Vibrionaceae*

*Vibrionaceae* are a large family of marine bacteria, many species of which are halophiles with an absolute requirement for NaCl, whereas others are halotolerant with an ability to survive in high salinity conditions. Within the Harveyi clade (members include *V. alginolyticus, V. antiquaries*, *V. azureus*, *V. campbellii, V. diabolicus, V. harveyi, V. jasicida, V. natriegens, V. owensii, V. parahaemolyticus*, and *V. rotiferianus*), most species are halophiles, as exemplified by *V. parahaemolyticus* [31]. *Vibrio parahaemolyticus* is an excellent model organism to study adaptations to changing salinity conditions since it can grow optimally in 0.5 M NaCl (~3% NaCl) and in salinities up to 1.5 M (~9%) on nutrient rich media [35]. In nutrient limited media (M9 minimal medium supplemented with glucose) where exogenous osmolytes are absent, *V. parahaemolyticus* cannot grow above 1 M NaCl (~6% NaCl) [35]. A *V. parahaemolyticus ectB* deletion mutant (Δ*ectB*), which cannot produce ectoine, cannot grow in M9Glucose 6% NaCl [36]. A substrate is considered a compatible solute for *V. parahaemolyticus* if it can rescue growth of the *ectB* mutant in M9Glucose 6% NaCl. Studies have shown that *V. parahaemolyticus* can uptake and utilize at least 14 different osmolytes, which include GB, DMG, DMSP, γ-amino-N-butyric acid (GABA), TMAO, ectoine, L-proline, L-glutamate, N-acetyl L-glutamine, glutathione, MOPS, creatine, trehalose, and octopine [20], [32], [35], [36], [77]. A compatible solute is considered more effective than another if it reduces the lag phase and/or increases the growth rate more. It was demonstrated that *V. parahaemolyticus* grown in 0.5 M NaCl allowed better survival in sublethal and lethal acid shock conditions, as well as persistence in high- and low-temperature conditions [150], [151]. Thus, growth in high salinity protects against other abiotic stresses. In the next sections, we will discuss what is known about the biosynthesis, transport and catabolism of compatible solute among *Vibrionaceae* and what can be predicted about these processes from genome mining analysis.

### 3.1 Compatible solute metabolism in Vibrio

Among *Vibrio*, ectoine biosynthesis genes are present in over half of the species present in the NCBI genome database as well as in members of the genera *Aliivibrio, Enterovibrio, Grimontia, Photobacterium* and *Salinivibrio* [36]. In *V. cholerae*, the *ectABC-asp* operon is present on chromosome II and is induced by high salinity. An *ectA* deletion mutant in this species cannot grow in >300 mM NaCl after 24 h*. Vibrio cholerae* does not contain the *betIBA* operon, and thus cannot utilize choline [39]. Interestingly, it was shown that in *V. cholerae*, L-proline and L-glutamate were not compatible solutes themselves, but served as substrates for ectoine biosynthesis [39]. *Vibrio anguillarum*, the causative agent of vibriosis in marine fish farms globally, can biosynthesize ectoine utilizing enzymes encoded by *ectABC-asp*. In this species, ectoine was essential for growth at low temperatures (5–18 °C) and the *ectA* gene was highly expressed in stationary phase cells [152]. It was also demonstrated that this species can produce GB from choline and GB was also accumulated in response to low temperatures [153]. Thus, it appears in *V. anguillarum*, the main function of GB and ectoine is as cryoprotectants. In *V. parahaemolyticus*, biosynthesis pathways for ectoine and GB, which cannot be catabolized in this species, are present making them *bona fide* compatible solutes. The expression of both the ectoine and GB biosynthesis genes were induced by NaCl [35]. No evidence was found that suggested the presence of an ectoine dioxygenase homolog (using EctD, KPQ27962 from *Halomonas* as a query sequence) in any *Vibrionaceae* genome available Thus, it appears Vibrionaceae cannot biosynthesize 5-hydroxyectoine. In *Salinivibrio* species, the *ectABC-asp* genes were present in all genomes suggesting this is an important feature of this genus. In this genus, the genes for trehalose biosynthesis were present but genes for its degradation were absent suggesting it can only be used as an osmolyte [154].

Catabolism of ectoine has not been demonstrated in *Vibrionaceae.* However, using EutD (WP_139526462.1), the ectoine hydrolase from *Halomonas elongata,* as a query sequence in BLAST analysis, we identified *eutD (doeA)* homologs in *Enterovibrio, Grimontia, Salinivibrio* and *Vibrio* species, including members of the Harveyi clade (*V. alginolyticus, V. antiquarius, V. diabolicus,* and *V. natriegens*). This suggests that ectoine can be used as a nutrient source by this group, each of which also contained the genes for ectoine biosynthesis. BLAST searches for homologs of EutA required for hydroxylectoine catabolism were present only in *V. diabolicus* (WP_145534461.1) from the Harveyi clade and three species of *Salinivibrio* (WP_077668456.1). A recent genome mining study of *Salinivibrio proteolyticus* M318 described the presence of a TRAP TeaABC transporter and the operon *doeABXCD* for ectoine catabolism in this species [155].

The *betIBA* operon is present in six genera of *Vibrionaceae* including a large number of *Vibrio* species with the notable exception of members of the Cholerae clade: *V. cholerae, V. mimicus, V. metoecus,* and *V. fluvialis* [35], [36]. In all *Vibrio* species that biosynthesize GB, the *betIBA* genes are in an operon with the *proXWV* genes, which encodes an ABC family transporter named ProU2 (Fig. 3) [31], [32], [35]. The co-occurrence of both ectoine and GB biosynthesis operons is prevalent in *Enterovibrio, Grimontia* and *Salinivibrio* species, and in some *Aliivibrio, Photobacterium* and *Vibrio* species. Within *Vibrio,* the presence of both operons appears to be conserved within specific clades such as Harveyi, Splendidus and Ordalii [31]. We recently reported the utilization of DMG, an intermediate in GB catabolism and biosynthesis, as an osmolyte in several *Vibrio* species, including *V. parahaemolyticus*, *V. cholerae, V. vulnificus*, *V. harveyi*, and *V. fluvialis* [77].

To date, GB catabolism has not been described in the *Vibrionaceae*. However, using the GB catabolism proteins (GbcA, DgcA and SoxB) present in *P. aeruginosa* as query sequences in BLAST homology searches, we identified homologs in several species of *Enterovibrio, Grimontia, Photobacterium*, and *Vibrio* in the NCBI genome database. In the genus *Vibrio*, these proteins were present in some strains of *V. fluvialis, V. gazogenes, V. mangrove, V. mytili, V. natriegens, V. palustris, V. ruber, V. spartinae, V. viridaestus,* and *V. xiamenensis*. This suggests that GB can be used as both an osmolyte and a nutrient source by this group. The first documentation of choline fermentation to TMA was from studies in *Vibrio cholinicus*, which was later reclassified as *Desulfovibrio desulfuricans* [156], [157]. We used the choline TMA-lyase (CutC, WP_012624983) from *D. desulfuricans* as a query sequence in BLAST searches of the NCBI genome database for homologs among *Vibrionaceae*. Choline TMA-lyase homologs were present in several species of *Photobacterium* and a single *Vibrio* species, *V. furnissii*, suggesting that choline can be fermented to TMA in a limited number of species.

As stated earlier, DMSP utilization as a compatible solute has not been extensively examined among bacteria. Recently, DMSP uptake and utilization as an osmoprotectant in *V. parahaemolyticus* was demonstrated and DMSP was effective as an osmolyte at nanomolar concentrations [37]. Furthermore, it was showed that *V. cholerae, V. vulnificus*, *V. harveyi*, and *V. fluvialis,* members of divergent clades, could also use DMSP as an osmolyte suggesting this ability is pervasive among *Vibrio* species [37]. These were the first studies to demonstrate a more extensive use of DMSP as an osmoprotectant among marine heterotrophic bacteria [37]. Algal blooms produce increased amounts of DMSP, as do corals, during warmer temperatures and *Vibrio* species associate with both. Thus, increased concentrations of DMSP may be an important driver in the ability of *Vibrio* species to proliferate and survive in these interactions. It will be of interest to determine whether *Vibrio* species that are pathogens of corals such as *V. coralliilyticus* can utilize DMSP as an osmo- and/or thermo-protectants.

Biosynthesis of DMSP has not been reported in Vibrionaceae and using DsyB from Labrenzia aggregate (AOR83342), required for DMSP production, we searched the NCBI genome database. A DsyB homolog was present in five Vibrio species with 97% query coverage, but low E-values 2e-18, and only 22% protein identity. Also, using methionine S-methyltransferase (MmtN) from *Alteromonadaceae* bacterium (WP_121467522.1) as a query sequence, we did not identify any similarity hits in *Vibrionaceae*. These analyses suggest DMSP biosynthesis is likely absent from *Vibrionaceae*.

To determine whether *Vibrio* species have the potential to catabolize DMSP, we used the DMSP lyases, DddD (accession number ACV84065 from *Halomonas* sp. HTNK1), DddP (KAF0171444 from *Rhodobacteraceae bacterium*), and DddK (WP_122473315 from *Pseudomonas syringae*) as query sequences in BLAST searches. DddD lyase is a class III CoA transferase that yields 3-hydroxypropionate and DMS from DMSP, whereas DddP and DddK are an M24 peptidase family protein and a cupin superfamily protein, respectively that yield acrylate and DMS from DMSP. We identified a number of DSMP lyase homologs with 98% query coverage, excellent E-values of 6.00E-171 and good protein identity of 54% in *Vibrio, Photobacterium, Grimontia* and *Enterovibrio* species. For example, a DddP homolog was present among a number of *Vibrio* species (Table S1) within a four gene cluster present in all species (Table S2). A DddD homology with an E-value of 0 and ~54% protein identity was present in *Photobacterium, Grimontia* and *Enterovibrio.* In *Enterovibrio norvegicus,* the DddD homolog was within a region previously described in *Halomonas* species that is required for DMSP catabolism (Table S3)*.* Overall, the data strongly suggests that *Vibrionaceae* has the ability to breakdown DMSP to DMS, which will need to be investigated further.

### 3.2 Compatible solute ABC family transporters in Vibrio

Bioinformatics analysis of the V. cholerae genome identified only a homolog of opuD, a GB BCCT transporter in Bacillus species, suggesting GB but not ectoine was a substrate for the transporter [39]. It was shown that *V. cholerae* wild type could uptake GB but not ectoine [39]. It was subsequently demonstrated that *V. cholerae* also possessed a PutP transporter that transported both L-proline and GB. In addition, OpuD was shown to transported L-proline [158]. However, a transcriptome analysis revealed that the expression of putP was not affected by salinity, but opuD expression increased with increasing salinity [159]. A V. vulnificus PutP homolog has been described and the *putP* gene was shown to be induced by NaCl and L-proline [160]. *Vibrio parahaemolyticus*, along with most members of the Harveyi clade, contain two compatible solute ABC transporters, namely ProU1 and ProU2, whereas species from the Cholerae clade (*V. cholerae, V. mimicus, V. metoecus*) lacked both of these transporters. Some ABC transporters uptake very specific substrates, while others indiscriminately uptake structurally diverse compounds [161]. ProU1 (VP1726-VP1728) is located on chromosome I encoded by *proVWX* operon and the ProU2 (VPA1109-VPA1111) is located on chromosome II and is in the operon *betIBA-proXWV* [31], [32], [35]. Analysis of a double mutant Δ*proU1/*Δ*proU2* showed this mutant had an extended lag phase in high osmolarity in defined medium indicating a defect in osmotolerance (Fig. S1).

The ProU systems in *V. parahaemolyticus* are unrelated to each other. ProU1 is similar to ProU of *E. coli* (~60% amino acid identity), and the ProU2 is more similar to OpuC from *Pseudomonas syringae* (~60% amino acid identity) [32], [35]*.* In *E. coli*, the ProU transporter was shown to be osmotically stimulated and mediated uptake of GB, proline betaine, choline, and other compounds [11], [145], [146], [147], [162], [163]. In *P. syringae*, OpuC, was shown to be involved primarily in the transport of GB and showed low affinity for choline [144]. The OpuC of *P. syringae* sensed changes in osmolarity and this function was mainly attributed to the presence of two cystathionine β-synthetase (CBS) domains arranged in tandem and located in the C-terminal portion of the ATP binding component [144]. In *V. parahaemolyticus* ProU1, the ATP binding component contains two CBS domains whereas ProU2 contains one CBS domain (Fig. S2). Growth analysis of a *V. parahaemolyticus* Δ*proU*1Δ*proU2* double mutant showed an extended lag phase in high salinity (M9G 6%NaCl) (Fig. S1). The addition of GB as an exogenous osmolyte removed the extended lag phase in the double mutant and it grew similar to wild type (Fig. S1). This indicates the defect in the double mutant was due to a defect in the osmotic stress response and not an overall growth defect. The CBS domains were deleted during the creation of double mutant Δ*proU*1Δ*proU2* strain, thus, it is possible that the extended lag time in the mutant could be due to a defect in osmosensing as a result of removing the CBS domains (Fig. S2).

### 3.3 BCCT family transporters in Vibrio

**Fig. 4:**
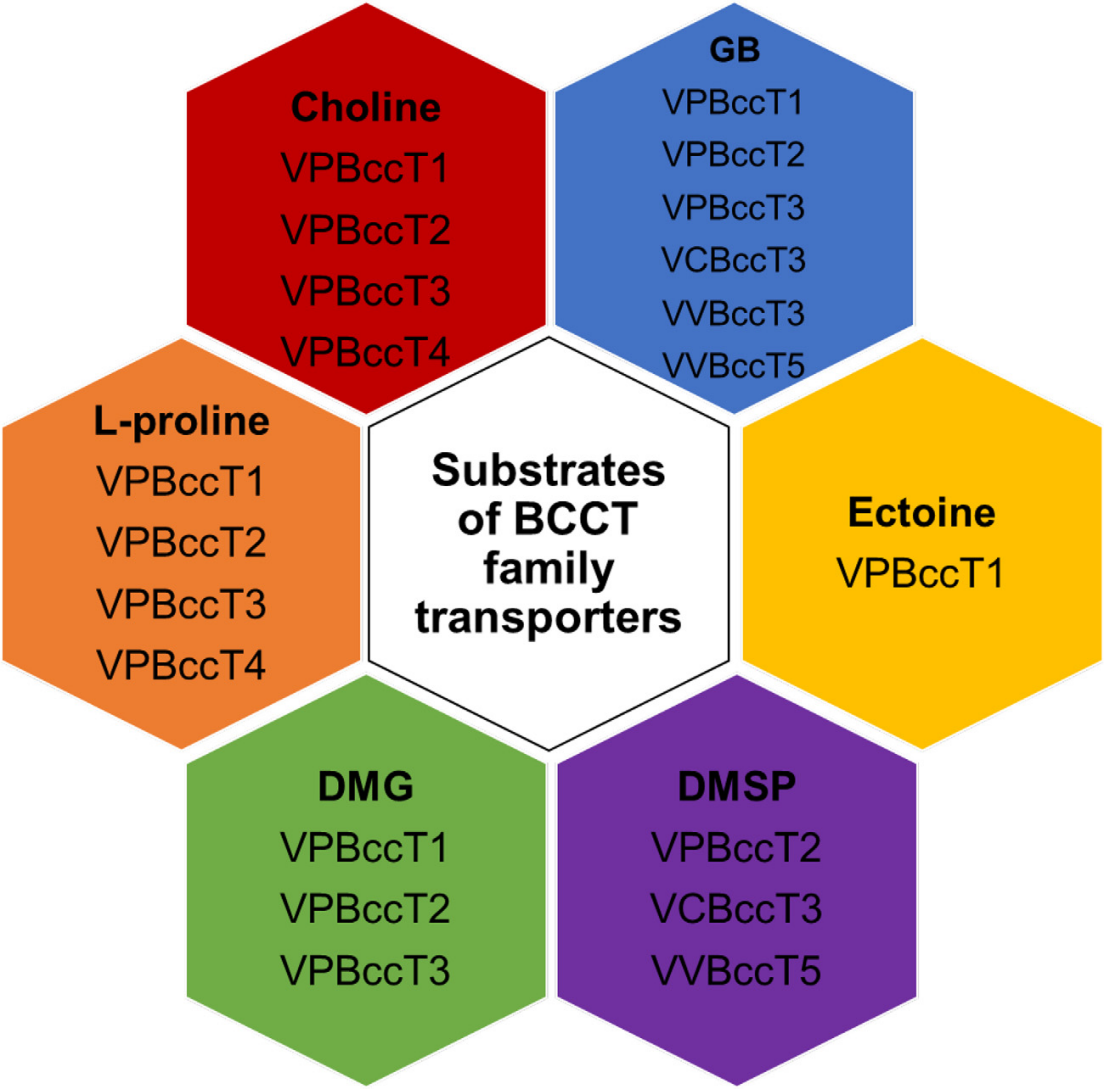
Experimentally confirmed substrates of BCCT family transporters from *V. parahaemolyticus* (VP), *V. cholerae* (VC) and *V. vulnificus* (VV).

In *V. parahaemolyticus,* four BCCTs encoded by VP1456 (*bccT1*), VP1723 (*bccT2*), VP1905 (*bccT3*), and VPA0356 (*bccT4*) are present in all strains (Fig. 3) [20]. All four BccT proteins are divergent from each other suggesting different substrate uptake abilities (Table S4). In *V. parahaemolyticus*, the BCCT family of transporters were shown to be crucial for the uptake of compatible solutes in this species. For example, BccT1 was shown to uptake GB, choline, DMG, L-proline, and ectoine efficiently (Fig. 4) [77]. BccT2 was demonstrated to transport glycine betaine, choline, DMG, DMSP and L-proline, while BccT3 transported GB, choline, DMG, and L-proline (Fig. 4). However, BccT4 could only uptake L-proline and choline (Fig. 4) [20], [77].

Within other members of the Harveyi clade, these four BccTs are generally present along with 2–3 additional BCCT family transporters [35], [37] (Fig. S3). Overall, species within the Harveyi clade can contain at least 11 divergent BCCT proteins [37]. Most species within this clade have 7 BCCTs, however, in *V. owensii* and *V. diabolicus* strains 8 BCCT carriers are present, whereas strains of *V. jasicida* have from 7 to 9 BCCT carriers [37]. In these species, the BCCTs were evenly distributed between the two chromosomes. The substrate specificity of each of these BCCTs is unknown, as is the reason why these species contain so many BCCTs. One could speculate that each BCCT has different substrate specificities and/or are expressed under different conditions or by different signaling mechanisms.

The BCCT1 transporter in *V. parahaemolyticus* is unusual in that it can uptake both compounds with methylated head groups (DMG, choline, and GB) and cyclic compounds (ectoine and L-proline) (Fig. S3). The GB binding pocket of BCCT transporters was shown to constitute the aromatic residues found in TM4 and TM8 (Fig. S4). It is known that these residues are highly conserved in BCCTs that uptake trimethylammonium compounds such as GB, L-carnitine and γ-butyrobetaine [24], [25], [140]. Studies have also showed that an additional tryptophan residue was present in TM8 outside of the binding pocket, and is believed to be involved in coordination of substrates during conformational changes that occur during transport [24], [25], [140].

Using bioinformatics analysis, the potential coordinating amino acid residues (Trp 203, Trp 208, Tyr 211, and Trp 384) in the substrate binding pocket for GB in the BccT1 protein were identified in *V. parahaemolyticus* [77]. Using site-directed mutagenesis, it was shown that a strain with all four of these amino acid residues mutated in the BccT1 protein resulted in abrogation of GB, DMG and ectoine transport. Additional site-directed mutagenesis analysis showed that three of the four residues were essential for ectoine uptake whereas only one of the residues was important for GB uptake. Overall, these studies demonstrated that GB, DMG and ectoine were coordinated in the same BccT1 binding pocket, but the residues required for coordination are strict for DMG and ectoine, while GB may be accommodated by alternate residues in single amino acid mutants [77]. Interestingly, homology analysis between BccT4 and BccT1 showed that BccT4 does not possess all four coordinating amino acid residues (Trp 203, Trp 208, Tyr 211, and Trp 384). The BccT4 protein lacked two of these residues, which may explain why this transporter had a limited substrate uptake ability (Fig. S4).

## 4 Regulation of compatible solute biosynthesis and transport in *Vibrionaceae*

Compatible solute biosynthesis and transporter systems are regulated via a combination of direct regulation and indirect regulation via exogenous compatible solutes and/or osmotic stress [4], [5], [23], [142], [164]. In this section we will discuss what is known about regulation of compatible solute systems in *Vibrionaceae*. The regulators of ectoine and GB biosynthesis will be discussed and how transporter expression is controlled will be examined.

### 4.1 Transcriptional regulators of ectoine biosynthesis in Vibrio

**Fig. 5:**
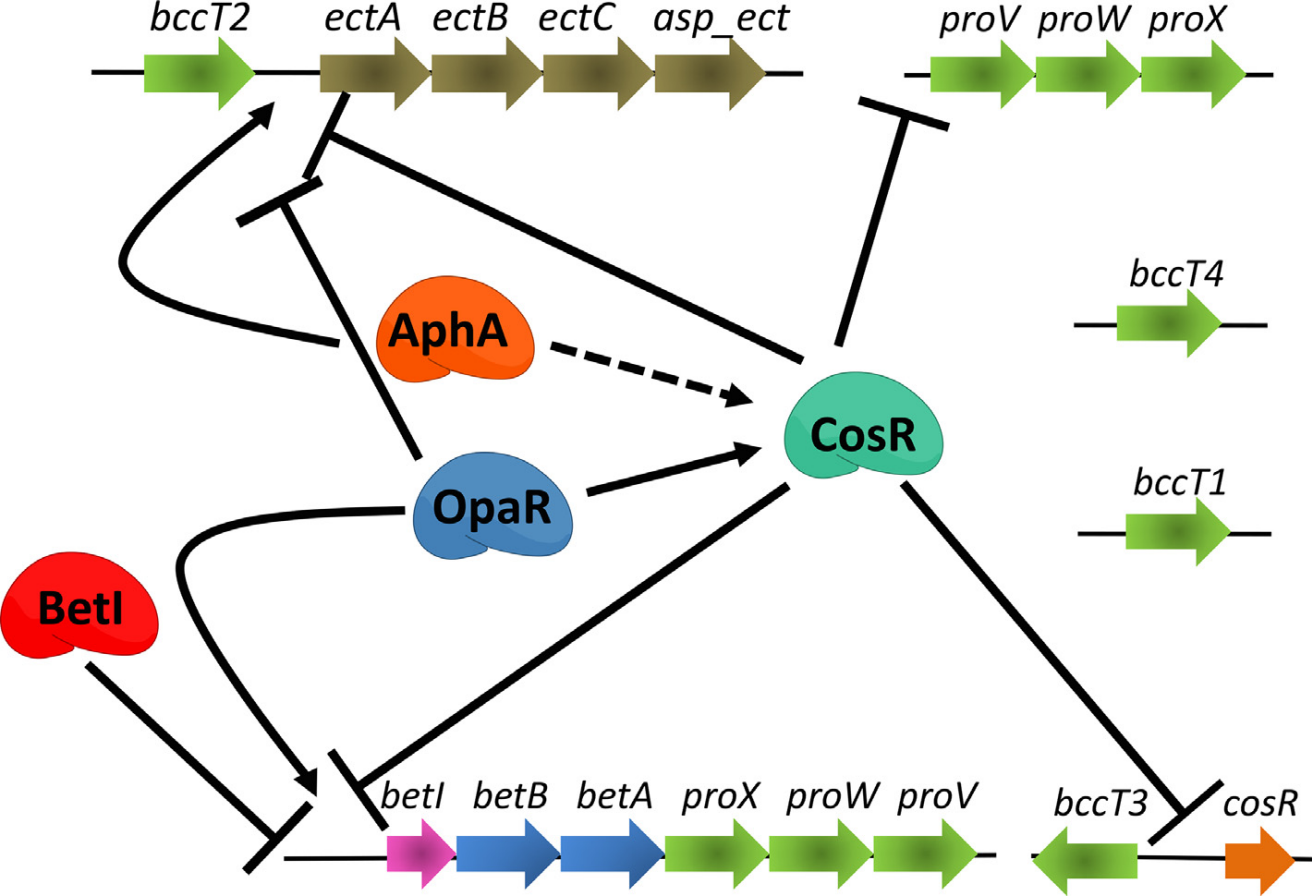
A model of CosR regulation of compatible solute systems in *Vibrio*. Solid arrows indicate direct positive regulation, dashed arrows, indirect positive regulation, solid hammers represent direct repression. The quorum sensing regulators OpaR and AphA were shown to directly and indirectly positively regulate CosR, respectively, and in addition directly regulate ectoine and glycine betaine biosynthesis operons.

The multiple antibiotic resistance (MarR)-type family of regulators are a large group of DNA-binding transcription factors with a helix-turn-helix domain characterized in bacteria. EctR1, a member of the MarR family, was identified as a local regulator of ectoine biosynthesis in the halotolerant methanotroph *Methylmicrobium alcaliphilum* [165]. EctR1 was shown to repress expression of the *ectABC-ask* operon in response to salinity [165]. In *V. cholerae*, a MarR-type repressor named CosR that shared 51% sequence identity to EctR1, was also shown to repress the *ectABC-asp* genes in low salinity conditions [166]. The *V. parahaemolyticus* homolog of *cosR* (VP1906) shares 70% identity with *cosR* from *V. cholerae* and was also shown to be a direct repressor of *ectABC-asp* in low salinity [43]. An in-frame deletion of *cosR* resulted in significant increased expression of *ectABC-asp* and purified CosR protein bound to the regulatory region of this operon in a concentration dependent manner. Reporter expression assays confirmed that CosR was a direct negative regulator of *ectABC-asp* in *V. parahaemolyticus* (Fig. 5) [43].

The role of quorum sensing master regulators OpaR and AphA in ectoine gene regulation was also investigated in *V. parahaemolyticus* [43]*.* Quorum sensing (QS) is a regulatory pathway that allows bacteria to coordinate gene expression in response to changes in cell density [167], [168], [169], [170]. In *Vibrio* species, LuxO is the major QS response regulator, and is an activator of the sigma factor RpoN that transcribes small quorum regulatory RNAs (named Qrr). Qrrs stabilize the translation of the low cell density master regulator AphA and destabilize the translation of the high cell density master regulator LuxR (named OpaR in *V. parahaemolyticus* and HapR in *V. cholerae*) [171]. Thus, in low cell density AphA is highly expressed and in high cell density OpaR is highly expressed. RNA-transcriptome analysis of a *luxO* mutant in *V. parahaemolyticus* in which *opaR* is constitutively expressed showed that the ectoine genes were repressed [172]. Further studies demonstrated that *ectABC-asp* operon was induced in an in-frame *opaR* deletion mutant [43]. These data suggested that OpaR was a negative regulator of *ectABC-asp*. DNA binding assays showed that purified OpaR bound to the *ectABC-asp* regulatory region indicating direct regulation by OpaR. In contrast, in an *aphA* deletion mutant, expression of *ectABC-asp* was repressed, which suggested AphA was a positive regulator [43]. DNA binding assays using purified AphA showed this protein bound upstream of *ectABC-asp* indicating direct positive regulation (Fig. 5). These data suggested that AphA is likely facilitating ectoine expression in early log phase when maximum growth is occurring and OpaR probably represses expression during entry into stationary phase when the requirement for ectoine production is likely diminished, as the cells approach a resting state and are no longer replicating [43].

Interestingly, bioinformatics analysis identified a putative OpaR binding site in the regulatory region of the *cosR* gene, located 180-bp to 199-bp upstream of the translation start [43]. Further analysis identified a feed-forward loop in which OpaR was a direct activator of *cosR*, while AphA was shown to be an indirect activator of *cosR* (Fig. 5). It was proposed that regulation of *ectABC-asp* via this feed-forward loop would allow for precise control of ectoine biosynthesis throughout the growth cycle to maximize fitness. Thus, at least three regulators are directly involved in transcriptional control of the ectoine biosynthesis genes and each of these regulators is probably affected by the other regulator since OpaR and AphA reciprocally repress each other, and both activate CosR [43].

### 4.2 Glycine betaine biosynthesis regulation in Vibrio

BetI is a direct transcriptional regulator of its own operon *betIBA* in *E. coli*, and expression analyses demonstrated that repression is removed in the presence of choline [68], [69]. This mechanism of regulation is conserved in both *V. harveyi* and *V. parahaemolyticus* where the operon is comprised of *betIBA-proXWV* [173], [174]. Direct regulation of compatible solutes transporters by the transcriptional regulator BetI was demonstrated in several species. In *E. coli*, BetI regulates the gene *betT,* a BCCT family transporter, which is divergently transcribed from *betIBA* [68], [69]. In *Acinetobacter baylyi*, which contains the *betIBA* operon, BetI was found to repress the transcription of two choline transporter genes, *betT1* and *betT2*. Unlike in *E. coli*, DNA binding assays revealed BetI released the regulatory region in the presence of choline. Levels of *betT1* and *betT2* were induced after growth in minimal media supplemented with choline over levels measured without choline, indicating repression by BetI is relieved [59]. In *Pseudomonas aeruginosa*, expression of *betT1* and *betT3* were upregulated in a *betI* mutant when grown in low salinity conditions, suggesting that BetI represses the expression of these transporter genes and the presence of choline relieved repression by BetI [19]. Nucleoid associated proteins have also been shown to play a direct role in compatible solute transporter regulation. A recent study showed that repression of the ProU promoter by H-NS in *E. coli* was relieved by IHF binding, which modifies the DNA secondary structure and activates transcription at the *proU* locus [175].

In *Vibrio harveyi*, a closely related species to *V. parahaemolyticus*, expression analyses demonstrated that the quorum sensing master regulator LuxR activated expression of the *betIBA-proXWV* operon [174]. A follow-up study demonstrated that IHF worked synergistically with LuxR to control *betIBA-proXWV* expression in this species [175]. In *V. parahaemolyticus,* it was shown that OpaR also directly regulated *betIBA-proXWV*, suggesting this mechanism of regulation could be widespread among *Vibrio* species (Fig. 5) [173]. A study in *Acinetobacter nosocomialis,* a Pseudomonadales*,* also showed that similar to the *Vibrio* species, the *betIBA* operon in this species was under the functional control of its QS master regulator AnoR [176]. Thus, quorum sensing appears to be an emerging mechanism of osmotic regulation in bacteria.

Recent data showed that CosR also played a key role in GB biosynthesis gene expression in *V. parahaemolyticus* [173]. A *cosR* deletion mutant was shown to have induced expression of *betIBA* at low salinity compared to wild-type [173]. DNA binding assays using purified CosR demonstrated direct binding to the *betIBA-proXWV* regulatory region. Further analysis using heterologous expression in *E. coli* GFP reporter assays, demonstrated that CosR directly repressed transcription of *betIBA-proXWV*. Phylogenetic analyses demonstrated that CosR was widespread and highly conserved within *Vibrio* suggesting that this could be a common mechanism of repression of both ectoine and GB gene expression. Interestingly in several *Vibrio* species, the *cosR* homolog was clustered with either the *ectABC-asp* operon and/or the *betIBA-proXWV* operon, which again suggests the importance of this regulator in controlling compatible solute biosynthesis*.* In four *Aliivibrio* species, *A. fischeri, A. finisterrensis, A. sifiae* and *A. wodanis* that contained *ectABC-asp*, an additional MarR-type regulator, which we named *ectR* was identified that clustered with these genes [173]. This suggests another MarR-type regulator could be required for regulation of ectoine biosynthesis in this genus*.* CosR was also shown to directly repress transcription of *bccT3* encoding the BCCT3 transporter and *proVWX*, which encodes ProU1. Overall these studies indicate that CosR is an important global regulator of the osmotic stress response in *Vibrionaceae* [173].

## 5 Summary and outlook

Many species of *Vibrionaceae* grow and survive in high salinity conditions. In this review, we focused on the roles of osmoregulated systems that included two biosynthesis systems (ectoine and GB) and six transporters (four BCCT-types and two ABC-types) present in this group of bacteria. Most of these systems are NaCl-inducible, but only the ectoine system was shown to be critical for growth at high osmolarity in a limited nutrient environment [20], [36]. This suggests that *de novo* osmolyte biosynthesis is likely critical for survival or at least important for fitness in high salt conditions when exogenous compatible solute are absent or limited. The *Vibrio* BCCT transporters appear to be critical for the uptake of most osmolytes and the ABC-type ProU transporters less so. In *Vibrio*, the BCCT transporters have a broad substrate range with BccT1 and BccT2 showing the most extensive substrate range, up-taking choline, GB, DMG, DMSP, ectoine, and L-proline (Fig. 4) [20], [37], [77], [173]. However, we do not know how these BccT transporters accommodate such diverse substrates in their binding pocket or why related BccT proteins from different species have different substrate specificities. For example, BccT3 from *V. cholerae* can transport DMSP whereas BccT3 from *V. vulnificus* and *V. parahaemolyticus* cannot. Our studies of the BccT proteins present in *Vibrio* have demonstrated that many species contain a large number of these proteins with some species having nine different BCCTs. Examination of the genomic context of BCCT genes suggests that they may be involved in more than compatible solute uptake but additionally may be involved in the uptake of substrates for metabolism.

A major area that that is less explored in marine heterotrophic bacteria such as *Vibrio* is the use of compatible solutes as nutrient sources. Compatible solutes are released into the environment by plant and animal cells, as well as bacteria during osmotic down shock and cells lysis. Our preliminary bioinformatics analysis predicts catabolism pathways for choline, ectoine, GB, DMG, L-carnitine, sarcosine and DMSP are present in *Vibrionaceae*. These gene clusters are phylogenetically widespread, present in several genera but sporadic in occurrence within each genera and species. It will be of interest to determine the evolutionary histories of these genes in this group and how the roles of osmoprotection and catabolism are balanced and controlled.

The regulation of compatible solute systems is only just beginning to be unraveled and the addition of quorum sensing regulators AphA and OpaR, as well as the MarR-type CosR regulator, to the list suggests much is to be learnt from studying marine halophiles. Another largely unexplored question in *Vibrionaceae,* is how species sense changes in osmolarity and orchestrate an osmoadaptive response. Analysis of the BCCT family proteins showed that unlike many BCCT transporters in Gram-positive bacteria, these proteins have short C-terminal domains. The C-terminal domains are believed to be important in osmosensing. In contrast, the BCCT proteins in *Vibrio* species have longer N-terminal domains that need to be investigated for a functional role.

## Declaration of Competing Interest

The authors declare that they have no known competing financial interests or personal relationships that could have appeared to influence the work reported in this paper.
