# Supplementary material for: Stressed out: Bacterial response to high salinity using compatible solute biosynthesis and uptake systems, lessons from Vibrionaceae
Source: Comput Struct Biotechnol J. 2021 Feb 1;19:1014–27. doi: 10.1016/j.csbj.2021.01.030 (PMC7876524; doi:10.1016/j.csbj.2021.01.030)
Supplement: Supplementary data 1 [file mmc1.pdf]

Supplementary Data

Computational and Structural Biotechnology Journal

Mini Review

Stressed out: Bacterial response to high salinity using compatible solute biosynthesis and uptake systems, lessons from *Vibrionaceae*

Gwendolyn J. Gregory and E. Fidelma Boyd*

Department of Biological Sciences, University of Delaware, Newark, DE, 19716

Table S1. List of *Vibrio* species with a homology of DddP (KAF0171444 from *Rhodobacteraceae bacterium)* required for dimethylsulfoniopropionate (DMSP) catabolism

**Species**	**Coverage**	**E value**	**Per. Ident**	**Acc. Len**	**Accession**
Vibrio fortis	98%	6.00E-171	53.97%	445	WP_032550763.1
Vibrio splendidus	98%	9.00E-171	54.20%	445	WP_060980452.1
Vibrio mediterranei	98%	1.00E-170	53.74%	445	WP_124941054.1
Vibrio fortis	98%	2.00E-170	53.51%	445	WP_150897716.1
Vibrio harveyi group	98%	3.00E-170	53.74%	445	WP_045372391.1
Vibrio coralliilyticus	98%	3.00E-170	53.74%	445	WP_172853848.1
Vibrio sp. Sal10	98%	3.00E-170	54.20%	445	WP_192890274.1
Vibrio	98%	5.00E-170	53.97%	445	WP_046224773.1
Vibrio nigripulchritu	98%	2.00E-169	52.83%	445	WP_045963477.1
Vibrio nigripulchritu	98%	3.00E-169	52.83%	445	WP_022593153.1
Vibrio orientalis	98%	3.00E-169	53.74%	445	WP_004418477.1

Table S2. Genome context of DddP homolog in *V. coralliilyticus* accession no. NZ_JABSMU010000002.1

Start	Stop	Strand	Locus tag	Protein product	Length	Protein Name
287271	288611	-	HRJ43_RS06820	WP_172853846.1	446	aspartate ammonia-lyase
288655	290160	-	HRJ43_RS06825	WP_172854017.1	501	aldehyde dehydrogenase
290377	291261	+	HRJ43_RS06830	WP_172853847.1	294	DddR LysR family regulator
291467	292486	+	HRJ43_RS06835	WP_022593154.1	339	aldo/keto reductase
293011	294348	+	HRJ43_RS06840	WP_172853848.1	445	**DddP M24 family metallopeptidase**
294358	295959	+	HRJ43_RS06845	WP_172853849.1	533	BCCT transporter
296370	296966	+	HRJ43_RS06850	WP_172853850.1	198	Integrase tyrosine recombinase

Table S3. Genome context of DddD homolog in *Enterovibrio norvegicus* accession no. NZ_AJYD02000038.1

Start	Stop	Strand	Locus tag	Protein product	Length	Protein Name
1157457	1157966	-	A1OO_RS13435	WP_016960442.1	169	gluconokinase
1158042	1159358	-	A1OO_RS13440	WP_016960443.1	438	TRAP transporter large permease subunit
1159351	1159911	-	A1OO_RS13445	WP_016960444.1	186	TRAP transporter small permease subunit
1159989	1161005	-	A1OO_RS13450	WP_017003322.1	338	TRAP transporter substrate-binding protein
1161407	1162900	-	A1OO_RS13455	WP_017003323.1	497	DddC CoA-acylating MMSA
1162918	1164078	-	A1OO_RS13460	WP_017003324.1	386	DddB iron-containing alcohol dehydrogenase
1164075	1165646	-	A1OO_RS13465	WP_016960448.1	523	DddT BCCT family transporter
1165926	1166855	+	A1OO_RS13470	WP_016960449.1	309	DddR LysR activator
1167012	1169528	+	A1OO_RS13475	WP_016960450.1	838	DddD Class III CoA transferase_3
1169622	1171052	-	A1OO_RS13480	WP_016960451.1	476	Na+/H+ antiporter NhaC
1171468	1171650	-	A1OO_RS23045	WP_083248059.1	60	DUF4260 family protein
1171631	1172071	-	A1OO_RS13485	WP_016960453.1	146	hypothetical protein
1172462	1172881	+	A1OO_RS13490	WP_016960454.1	139	D-ribose pyranase

MMSA = methylmalonate-semialdehyde dehydrogenase

Table S4. Amino acid identity of the betaine-carnitine-choline transporters (BCCTs) in *V. parahaemolyticus*. VP1456 (BccT1), VP1723 (BccT2), VP1905 (BccT3), andVPA0356 (BccT4), *V. cholerae* OpuD (VC1279/BccT3), and *Escherichia coli* ProP (MSF), PutP (SLC5-SLC-6 protein), and BetT (BCCT) were compared

	% Identity							
**ORF**	**VP1456**	**VP1723**	**VP1905**	**VPA0356**	**VC1279**	**ProP**	**PutP**	**BetT**
**VP1456**	100	50.5	67.8	30	67.8	43.5	25.5	37.5
**VP1723**	50.5	100	52.7	29.6	49.1	26.8	27.5	35.2
**VP1905**	67.8	52.7	100	30.5	81.8	38.2	23.7	35.3
**VPA0356**	30	29.6	30.5	100	30.5	36.8	58.3	30

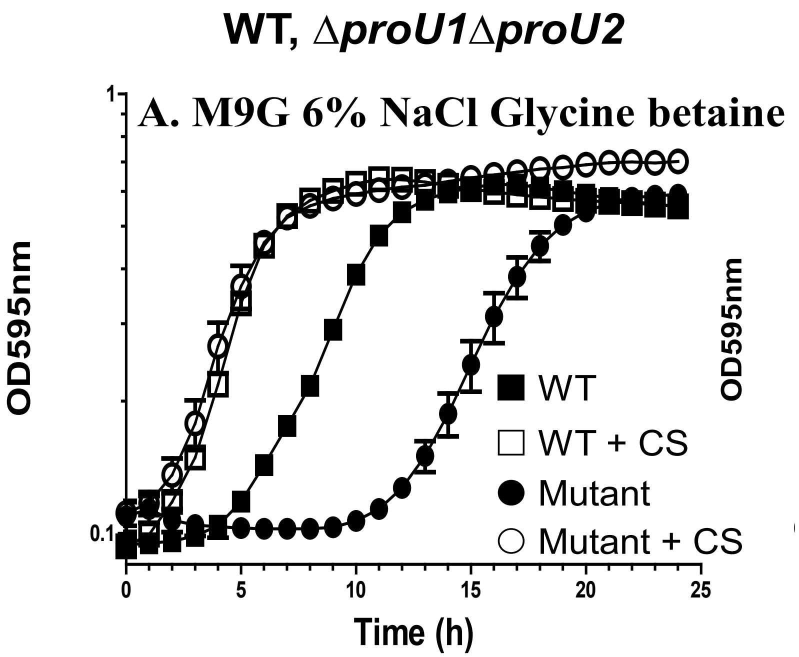

Fig. S1. Growth analyses of *V. parahaemolyticus* wild type (WT) and Δ*proU2*Δ*proU1* double mutant. M9G 6%NaCl+ Glycine betaine (CS). Analyses were performed in triplicate and the data are shown as pooled of two biological replicates.

A Conserved domains of ProV(VP1726) of ProU1

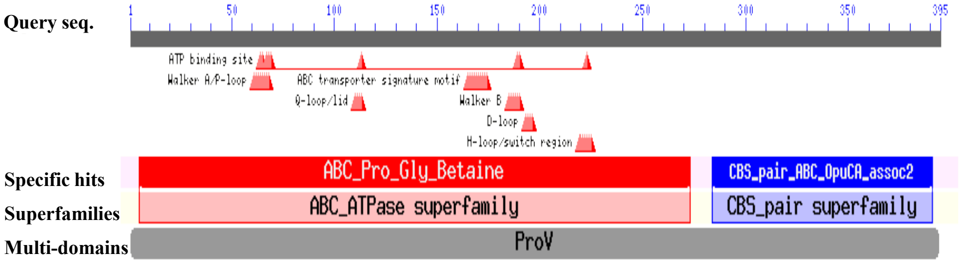

B Conserved domains of ProV (VPA1109) of ProU2

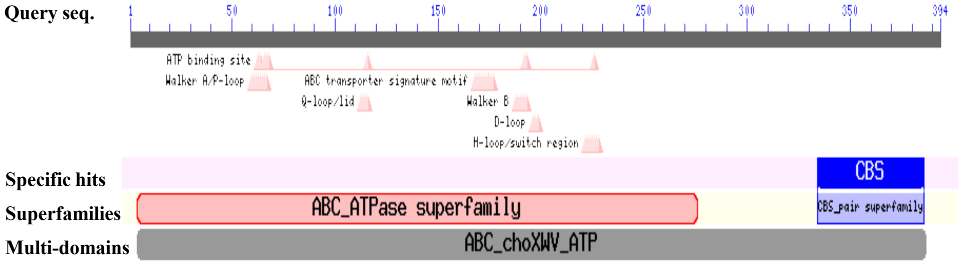

Fig. S2. Conserved domains of the ProV subunits of the ProU systems. NCBI Conserved Domain (CDD) Database for the functional annotation of proteins was used for CDD search [203-205].**A**. ATP-binding subunit ProV (VP1726) of ProU1.**B**. ATP-binding subunit ProV (VPA1109) of ProU2.

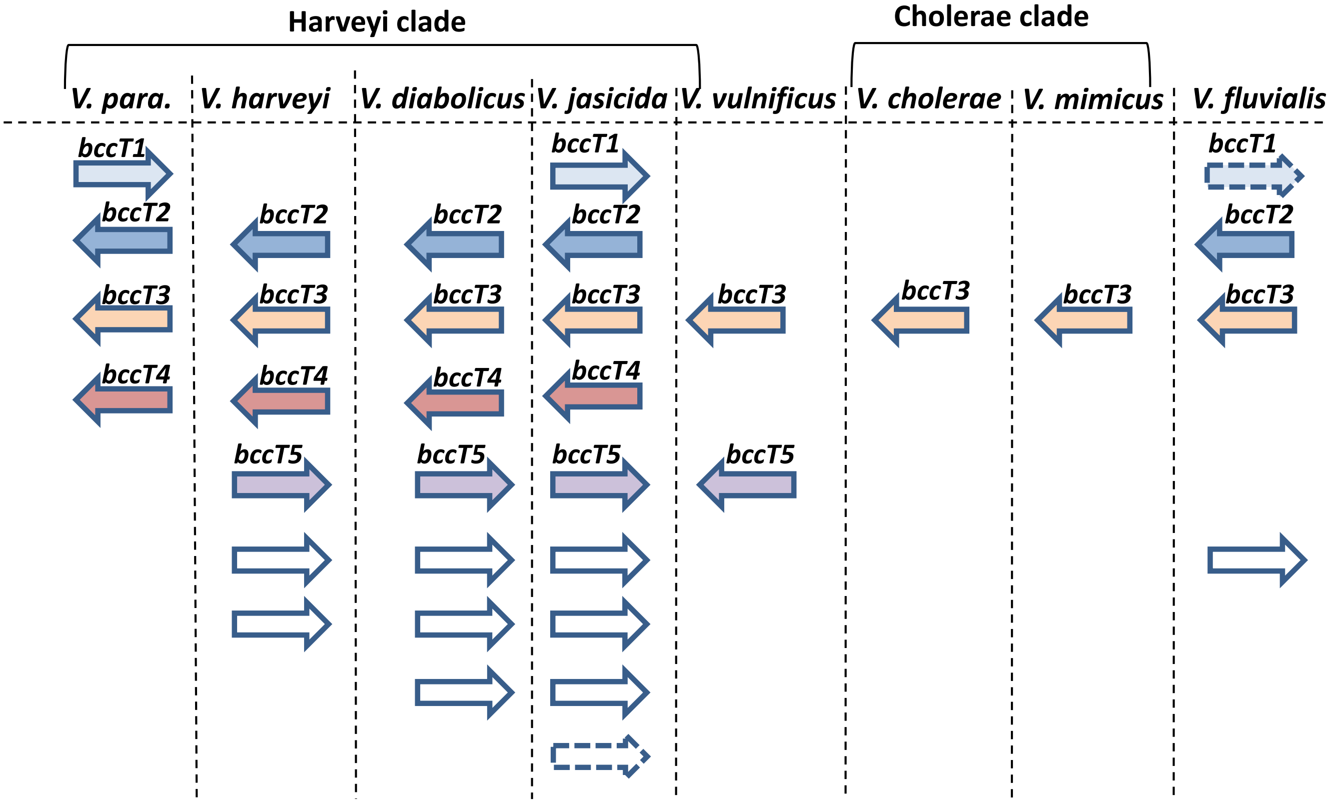

Fig. S3. Distribution of BCCT transporters among select *Vibrio* species. Arrow indicate the direction of transcription and dotted arrow indicates not present in all strains
